# Medical Congress

**Published:** 1890-02

**Authors:** 


### Medical Congress.

The members of the congress first mentioned, manifested a marked unanimity of opinion in regard to the relation of insanity to the consumption of alcohol, and the following resolutions or views were adopted :

1. The increased consumption of alcohol is one of the principal causes of the development of crime and insanity.
2. A diminution of the number of drinking places being one of the means of reducing the consumption of alcohol, this congress is of the opinion that governments should take measures to restrict the number of dramshops.

Opinions were also expressed that crimes committed during the delirium of inebriety should not entail full responsibility but, on the other hand, that society should be protected against such drunkards by their being subject to sequestration in suitable retreats.

Whatever may be one's personal opinion, the expression of the above views, coming as they do from an intelligent body of men, must carry emphatic weight. It must be borne in mind that the members of this congress probably never contemplated prohibitory legislation, that they live in countries where in some form alcohol is, as a rule, a part of the dietary, that in no way can the cry of fanaticism be raised, and that in all probability the great majority of them use alcoholics more or less themselves. This lends added importance to their action.

It is probably well within the limits of facts to consider the abuse of alcohol as not only capable of causing insanity, but as being a frequent cause of this the most distressing and wide-reaching calamity that can befall an individual or a family, and which imposes a heavy burden of tax and responsibility upon the community at large.

